# The Impact of RNA Interference in Tick Research

**DOI:** 10.3390/pathogens11080827

**Published:** 2022-07-23

**Authors:** José de la Fuente, Katherine M. Kocan

**Affiliations:** 1 SaBio, Instituto de Investigación en Recursos Cinegéticos IREC-CSIC-UCLM-JCCM, Ronda de Toledo s/n, 13005 Ciudad Real, Spain; 2 The Department of Veterinary Pathobiology, Center for Veterinary Health Sciences, Oklahoma State University, Stillwater, OK 74078, USA; katherine.kocan@okstate.edu

**Keywords:** RNAi, tick, vaccine, CRISPR, arthropod, pathogens

## Abstract

Over the past two decades, RNA interference (RNAi) in ticks, in combination with omics technologies, have greatly advanced the discovery of tick gene and molecular function. While mechanisms of RNAi were initially elucidated in plants, fungi, and nematodes, the classic 2002 study by Aljamali et al. was the first to demonstrate RNAi gene silencing in ticks. Subsequently, applications of RNAi have led to the discovery of genes that impact tick function and tick-host-pathogen interactions. RNAi will continue to lead to the discovery of an array of tick genes and molecules suitable for the development of vaccines and/or pharmacologic approaches for tick control and the prevention of pathogen transmission.

## 1. Introduction

RNA interference (RNAi) is a molecular methodology that, in combination with recent omics technologies, has substantially advanced the understanding of tick gene function [1,2,3]. RNAi is effected by the introduction of double-stranded RNA (dsRNA) homologs to specific messenger RNA (mRNA), resulting in sequence-specific degradation, thereby interfering with gene expression, causing a subsequent loss of gene function.

The RNAi method used today evolved from studies on cellular phenomena identified initially in 1990 by Napoli et al. [4] in petunia plants. In this study, the authors hypothesized that the introduction of a chimeric gene would result in reversible co-suppression of homologous genes in trans. A similar phenomenon was subsequently reported in the fungus *Neurospora crassa*, which proposed that the introduction of homologous RNA would cause quelling of a target gene [5]. Subsequently, RNAi was first reported in 1995 in the nematode *Caenorhabditis elegans* [6].

In 1998, a pioneering study by Fire et al. [7] elucidated the mechanisms by which dsRNA impacted the phenomena of co-suppression in plants [4], quelling in fungi [5], and RNAi in nematodes [6]. Further key advances in RNAi research included evidence of parent-to-offspring transmission in *C. elegans* [8,9], identification of small interfering RNAs (siRNAs) as stable RNAi intermediates [10,11,12,13], siRNA-mediated silencing of endogenous and heterologous genes in mammalian cells [14], and identification of enzymes and other components of the RNA-induced silencing complex (RISC) [15,16,17].

The first report of RNAi in ticks in 2002 by Majd N. Aljamali, John R. Sauer, and Richard C. Essenberg from Oklahoma State University (Stillwater, OK, USA) [18], based on these previous studies, was a fundamentally important advance in the study of ticks. RNAi in ticks led to the elucidation of tick gene function and the molecular interactions between ticks and pathogens required for pathogen development and transmission. These studies allowed for the identification of tick genes for use in the development of novel interventions for the control of tick infestations and tick-borne diseases [19].

## 2. Discovery/Methodology

The classic proof-of-concept study of dsRNA-mediated RNAi in ticks by Aljamali et al. [18] was done using *Amblyomma americanum* female ticks. In this study, dsRNA targeted to the histamine binding protein (HBP) by RNAi was incubated in vitro with extracted tick salivary glands or injected into female ticks. The incubation and injection of this specific dsRNA caused a reduction in HBP mRNA levels impacting tick feeding, most likely due to higher histamine concentrations at the feeding site [18].

**Figure 1:**
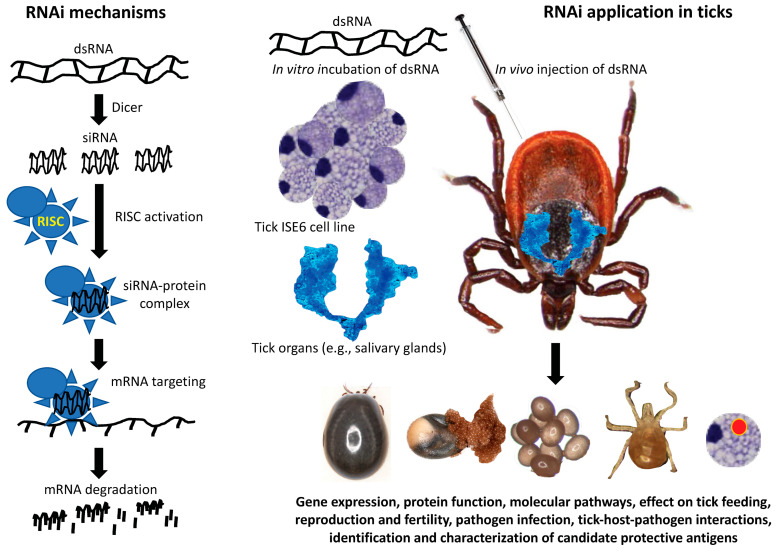
Model of the mechanism of RNAi applications in tick biology. Both model and applications were inspired in the classic paper by Aljamali et al., 2002 [18]. *Ixodes scapularis* tick and ISE6 cell images are courtesy of the authors.

These and other methodologies subsequently developed for RNAi in ticks confirmed previous reports using other species, provided methods for the study of tick gene function, and also led to development of applications for studies on other arthropod ectoparasites (Figure 1). 

RNAi in ticks is induced with endogenously present or exogenously introduced dsRNA cleaved to produce siRNAs (21–25 bp) by the ATP-dependent RNase III-like enzyme Dicer. The siRNAs then recruit and activate RISC resulting in unwinds of the siRNA in the siRNA-protein complex. Each strand of the siRNA binds to complementary sequences with activated RISC binding to the targeted RNA, cleaving it and resulting in mRNA degradation. For RNAi in ticks, dsRNA can either be injected in vivo into live ticks or incubated with tick tissues (e.g., salivary glands) or cultured tick cells [20]. The assessment of the effect of RNAi can subsequently be done by analysis of gene mRNA expression and by the impact on the target gene protein function. Additionally, the effect of RNAi on molecular pathways, tick feeding, reproduction and fertility, pathogen infection, and tick-host-pathogen interactions can be evaluated. Through this methodology, candidate protective antigens can be identified, studied, and used for the discovery of novel antigens for vaccine development or for use in other tick interventions. 

## 3. Impact

**Figure 2:**
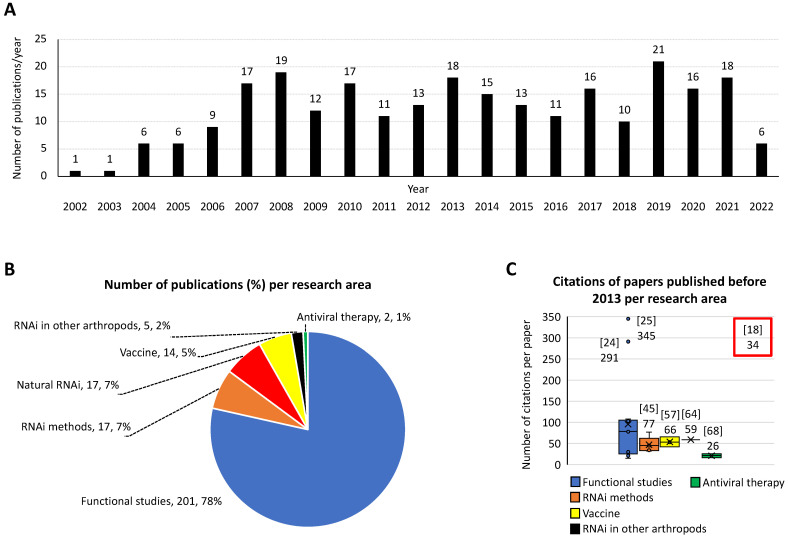
Scientometric and bibliometric analyses of RNAi applications in ticks and other arthropod species. Analyses were conducted based on publications identified in PubMed (https://www.ncbi.nlm.nih.gov; accessed on 10 June 2022) using search with “RNA” and “interference” and “tick” terms on 10 June 2022. (**A**) Number of publications per year. (**B**) Number of publications and percentage per research area. (**C**) Number of citations per paper published before 2013 (in the first decade after the classic paper by Aljamali et al., 2002 [18] was published) per research area. Reference to the papers with highest citation score with corresponding number of citations at Web of Science (https://www.webofscience.com) accessed on 10 June 2022, are shown. The citations of the classic paper by Aljamali et al., 2002 [18], highlighted in this commentary, also shown.

The development and validation of RNAi in ticks has impacted scientific research by advancing studies on a wide variety of arthropod species, ranging from basic biology to biotechnological studies [19]. Using scientometric and bibliometric analyses, 256 publications were identified in PubMed (https://www.ncbi.nlm.nih.gov) searching with “RNA”, “interference”, and “tick” terms on 10 June 2022 (Figure 2A). As shown in this analysis, the number of publications per year increased from one in 2002, the year of the publication of first classic paper [18], to over ten publications per year from 2007–present (Figure 2A).

While the application of RNAi has advanced research in several areas (Figure 2B,C), most of the investigations (201 publications, 78%) with the highest citation scores were focused on tick functional studies. Furthermore, research on naturally occurring RNAi mechanisms and RNAi methods represented 7% (17 publications each) of the total number of publications. Other research areas included vaccines for the control of tick infestations and tick-borne pathogens (14 publications, 5%), RNAi in other arthropods (5, 2%), and antiviral therapy (2, 1%). The examples below illustrate the impact of RNAi on various areas of tick research. 

RNAi has been used to address many aspects of tick biology by enabling the functional analysis of individual genes or combinations of genes. Soon after the publication of the classic paper of this commentary, Aljamali et al. [21] reported a silencing of the histamine binding protein (HBP) in *A. americanum* in vivo and salivary glands, resulting in reduced histamine binding capacity and altered tick feeding. A second paper by Narasimhan et al. 2004 [22] reported a disruption of the anticoagulation response in *I. scapularis* by silencing the salivary gland gene that expresses the anticoagulant Salp14. In 2006, de la Fuente et al. demonstrated the impact of the silencing of a single gene (subolesin), resulting in sterile male ticks that were unable to mate successfully with female ticks, thus inhibiting completion of female engorgement and oviposition [23]. Collectively, these initial RNAi functional studies in ticks demonstrated the relevance of this methodology for enhancing the understanding of tick biology. Additional subsequent studies have included the knockdown of different tick genes for the characterization of multiple biological pathways involved in tick-host-pathogen interactions [24,25,26,27,28,29,30,31,32,33,34,35,36,37,38,39,40,41]. In many studies, the results were used to propose new candidate protective antigens for vaccine development or for use as pharmaceutical targets [23,33,34,35,37].

RNA viruses were studied early [42], and RNAi is the major antiviral mechanism against arboviruses in arthropod vectors. As in other organisms, this natural antiviral response was also subsequently characterized in ticks [43,44,45]. 

RNAi methodology evolved from the original in vitro incubation of dsRNA with tick salivary glands and from in vivo studies in live female ticks injected with dsRNA [18,20]. The variety of methodologies for tick RNAi subsequently included, in chronological order, (a) RNAi in cultured tick cells [46], (b) transovarial RNAi [47], (c) in vitro feeding assays for hard ticks [48], (d) dsRNA electroporation in tick eggs and nymphs [49], (e) cytoplasmic RNA viruses as vehicles for the efficient delivery of therapeutic small RNAs [50], (f) dual luciferase reporter systems for optimization of RNAi [51], (g) non-invasive delivery of dsRNA into de-waxed tick eggs by electroporation [52], (h) tick immersion in dsRNA [53], (i) liposome mediated dsRNA delivery [54], (j) delivery of a genetically marked *Serratia* AS1 for RNAi [55], (k) functional RNAi analyses using tick organ cultures [56], and (l) cationic glycopolyelectrolytes for RNAi in tick cells [57].

RNAi has also been used to identify and characterize candidate protective antigens for the control of tick infestations and tick-borne pathogens [23,58,59,60,61,62]. In this approach, the selection of protective antigens can be done prior to testing in animal vaccine trials. 

Following validation of RNAi in ticks [18], the methodologies were also used in other arthropod species, including the fruit fly *Drosophila melanogaster* [63], salmon louse *Lepeophtheirus salmonis* [64], honeybee mite *Varroa destructor* [65,66], and mosquito *Anopheles arabiensis* [67]. These studies were focused on the characterization of the function of proteins in the biological processes of the arthropod life cycle, and on pathogen infection and transmission. 

The proposal of RNAi-based therapeutics for controlling the tick-borne encephalitis virus (TBE) [68] and other flavivirus [69] infections was also based on the development of antisense-based approaches derived from RNAi research in ticks and other arthropod species.

Recently, RNAi has been incorporated into research on the Alpha-Gal Syndrome (AGS), an IgE-mediated, delayed-type allergic reaction in response to the oligosaccharide galactose-α-1,3-galactose (α-gal). Alpha-gal is present in tick biomolecules and injected into humans during tick feeding [70]. 

In conclusion, RNAi is the leading gene expression manipulation tool in arthropods followed by gene editing via the bacterial type II Clustered Regularly Interspaced Short Palindromic Repeats and associated protein 9 system (CRISPR-Cas9) [71]. Characterization of the microbiota composition in ticks and other arthropods may also provide targets for the production of modified bacteria using paratransgenesis or RNAi [54,72,73,74]. The recent report of the successful application of CRISPR-Cas9 in ticks [75] provides an opportunity to combine these methodologies for the manipulation of tick gene expression and the development of paratransgenic interventions for the control of tick infestations and transmission of tick-borne pathogens.

## Data Availability

Not applicable.
